# Toward a more physiologically and evolutionarily relevant definition of metal hyperaccumulation in plants

**DOI:** 10.3389/fpls.2015.00033

**Published:** 2015-01-30

**Authors:** Eric W. Goolsby, Chase M. Mason

**Affiliations:** ^1^Department of Plant Biology, University of GeorgiaAthens, GA, USA; ^2^Interdisciplinary Toxicology Program, University of GeorgiaAthens, GA, USA

**Keywords:** hyperaccumulation, tolerance, metals, metalloids, physiology, evolution, traits

## Introduction

More than 500 plant species have demonstrated the ability to absorb metals or metalloids from soils and concentrate them at extremely high levels in leaf tissues (van der Ent et al., 2013; Cappa and Pilon-Smits, 2014). This phenomenon, known as hyperaccumulation, has profound ecological implications including herbivory deterrence, trophic transfer of metals, and modifications of soil chemistry (Boyd, 2004; Rascio and Navari-Izzo, 2011; Fones and Preston, 2013). Over the past few decades, many attempts have been made to develop an unambiguous, standardized definition of metal hyperaccumulation in plants (Baker and Whiting, 2002; van der Ent et al., 2013; Pollard et al., 2014). Several general recommendations and guidelines for standardizing hyperaccumulation research have been put forward recently, not all of which are compatible with the examination of hyperaccumulation from physiological, genetic, and evolutionary perspectives. Here we highlight several key issues with previous guidelines, and propose a refined definition that is more reflective of both the genetic and physiological mechanisms underlying hyperaccumulation and the evolutionary history of this phenomenon.

## Recent recommendations and guidelines

### Greenhouse studies employing amended soils are less valid than those using natural soils

Several sources have suggested that using artificially amended or “spiked” soils in greenhouse studies of hyperaccumulation is undesirable for both identifying hyperaccumulators and understanding their physiology (Chaney et al., 2010; van der Ent et al., 2013; Pollard et al., 2014). The main criticisms of the use of amended soils are that (1) virtually any plant can be induced to take up metals under very high soil concentrations, though most doing so would not survive to reproduction (Baker, 1981), and (2) that amended soils do not mimic the conditions of natural soils (van der Ent et al., 2013). While we agree that passive hyperaccumulation (induced by extremely high soil concentrations) should be distinguished from active hyperaccumulation (achievable under lower soil concentrations), soil amendments provide the ability to experimentally manipulate soil metal concentrations and thus identify minimum concentrations at which hyperaccumulation can be achieved, as well as maximum concentrations species can tolerate (Pollard, 2000). This teasing apart of tolerance and hyperaccumulation is key to the understanding of hyperaccumulator evolution, as we will outline below. Additionally, amended soils are the only way to experimentally explore the effects of other soil characteristics (e.g., pH, cation exchange capacity, etc.) on hyperaccumulation, as well as determine whether hyperaccumulators of one metal also have the ability to take up others. The claim that amended soils don't mimic natural ones makes little sense, as soils are incredibly heterogenous and anthropogenic sources of contamination, including mine tailings and chemical spills, have given rise to new hyperaccumulators. There is no inherent quality of “natural” soils that makes them superior for the purposes of hyperaccumulator investigations. Recommending greenhouse studies of hyperaccumulation use only natural soils presents a variety of problems, chief among them that using only natural soils in pots can limit the total amount of metal available to the plant given the constrained soil volume and lack of replenishment of the rooting zone that would typically occur in the field. A pot-grown hyperaccumulator will often exhaust the available metals in the soil rather quickly, resulting in dilution of tissue metal concentration as the plant continues to grow. In this way, depending on the natural soil metal concentration, true hyperaccumulators can fail to be detected, especially if plants are grown to reproduction as recommended to confirm tolerance, and are thus large enough that reaching hyperaccumulation thresholds in tissue may be mathematically impossible.

### Only species that exhibit hyperaccumulation in natural populations are appropriately defined as hyperaccumulators

Hyperaccumulation has been defined by several sources from an ecological perspective (van der Ent et al., 2013; Pollard et al., 2014), with the recommendation that species only be recognized as hyperaccumulators if they have been found to achieve threshold leaf metal concentrations in natural populations in the field. This naturalistic definition ignores hyperaccumulation as a physiological trait, and is not consistent with how plant physiological traits are typically characterized. For instance, abiotic stress tolerances are defined by inherent capacities, not solely by the demonstration of these capacities in the field. Many salt, drought, fire, and frost tolerant species can also grow where these stresses are absent or infrequent, despite predictions that energetic tradeoffs would result in loss of tolerance (e.g., the cabbage palm *Sabal palmetto* is tolerant of salt, drought, fire, and frost, but also occurs in non-saline mesic habitats with frequent standing freshwater which never burn, and spans a range from areas of North Carolina with annual frost to the frost-free tropical Caribbean). Likewise, traits such as pest or pathogen resistance are often not observable until plants are exposed, and one would not define these resistances as not existing simply because a population does not happen to encounter specific pests or pathogens where it grows. In fact, several crop pest resistance traits have been derived from wild relatives that grow where the pest has never been present (e.g., broomrape resistance in sunflower; Ruso et al., 1996), indicating that resistance is often a by-product of another process. In a similar vein, hyperaccumulation is an intrinsic ability of a plant based on the presence of appropriate ion pumps, transporters, and other physiological mechanisms (Rascio and Navari-Izzo, 2011), and this ability exists regardless of the presence of metals in the soil in which a plant currently grows. It is quite likely that biotic and other non-edaphic factors drive some hyperaccumulators to occur on soils without sufficient metal concentrations to achieve hyperaccumulation thresholds, despite the fact that these species still retain the ability to hyperaccumulate and would show the phenotype if grown in amended soils or if the vagaries of nature returned them to metal-rich soils (Boyd and Martens, 1998). Additionally, hyperaccumulation may in some cases be the result of inadvertent metal uptake as a byproduct of affinity for other elements, either other metals or soil nutrients (Boyd and Martens, 1998). Accordingly, hyperaccumulation ability for a specific metal could evolve without a species ever interacting with substantial quantities of that specific metal, especially given that for some metals hyperaccumulation has been shown to be a single-gene trait (e.g., Hanikenne et al., 2008). Given a simple genetic basis of hyperaccumulation, it is even possible that genetic drift resulting in increased expression of certain classes of fundamental transporter proteins present in all plants could result in the evolution of hyperaccumulation without tolerance.

Restricting the definition of hyperaccumulation to only include species exhibiting threshold leaf metal concentrations in natural populations is inappropriate, as this definition links the classification of a physiological trait with the physical location of plants, and is especially troubling as it excludes all known domesticated species with hyperaccumulation ability as these crops do not form natural populations (e.g., sunflower, rice, corn, canola). A naturalistic definition also ignores evolutionary history, as it excludes hyperaccumulators that historically occupied soils with high metal concentrations but now do not (for whatever reasons), as well as all lineages retaining hyperaccumulation ability as an ancestral trait or exaptation, or that have evolved hyperaccumulation outside of the context of metalliferous soils by drift or a byproduct of other processes. Even worse, by assuming that hyperaccumulation ability could only evolve in response to metal-rich environments, current naturalistic definitions approach adaptationism (Gould and Lewontin, 1979). The possibility of hyperaccumulation as a widespread latent trait is supported by the broad distribution of hyperaccumulators across the angiosperm phylogeny (Kraemer, 2010; Cappa and Pilon-Smits, 2014). This issue is of particular importance given that the discovery of hyperaccumulation ability in domesticated plants and their wild relatives can have valuable potential for phytoremediation, biofortification, and other technological applications.

## An explicitly physiological and evolutionary perspective

**Figure 1:**
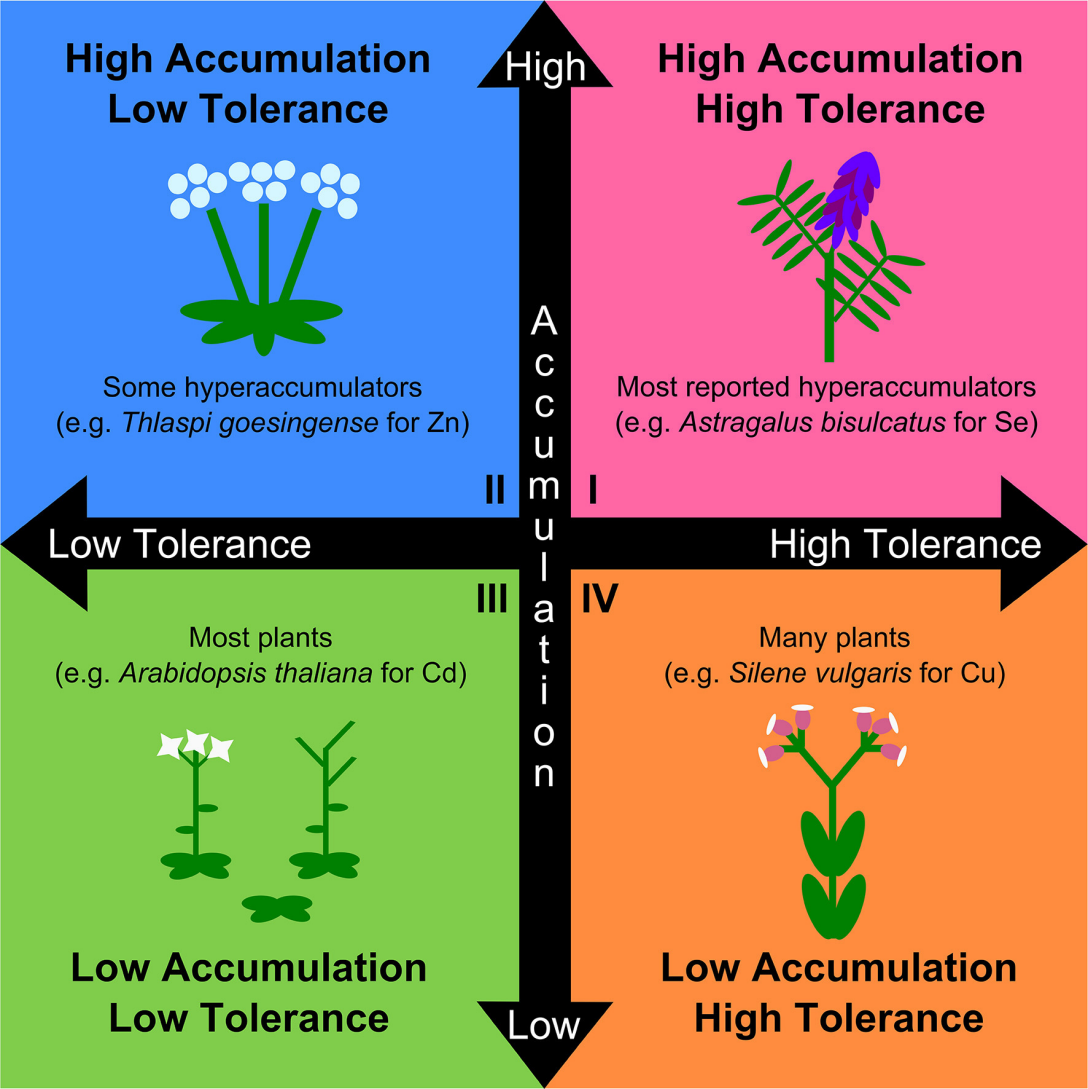
**A more physiologically and evolutionarily relevant conceptual framework for the study of variation in the distinct physiological traits of metal accumulation and tolerance**. Both traits are continuous, and plant phenotypes span a wide range of combinations of both traits. Traditional hyperaccumulators are those with both high accumulation and high tolerance (**Quadrant I**; e.g., *Astragalus bisculcatus* for selenium; (El Mehdawi et al., 2011)). Species with high accumulation of specific metals, but low tolerance to those metals, are also known to exist but are excluded from the naturalistic definition of hyperaccumulation (**Quadrant II**; e.g., *Thlaspi goesingense* for zinc; (Lombi et al., 2000)). Most plants are neither tolerant nor accumulate (**Quadrant III**; e.g., *Arabidopsis thaliana* for cadmium; (Hanikenne et al., 2008)), though many species are known to be tolerant of metal exposure while not accumulating (**Quadrant IV**; e.g., *Silene vulgaris* for copper; (van Hoof et al., 2001)). Both groups **(III)** and **(IV)** are typically collapsed together as non-hyperaccumulators, which is accurate, but fails to capture the important distinction and its relevance to the colonization of metal-rich soils and likely importance in hyperaccumulator evolution. Explicitly considering both tolerance and accumulation as separate traits is key to the study of hyperaccumulator physiology and evolution.

The naturalistic definition of hyperaccumulation attempts to maximize ecological relevance, but at the expense of physiological, genetic, and evolutionary relevance. Given the trajectory of hyperaccumulator research toward evolutionary questions (e.g., adaptive hypotheses such as the elemental defense hypothesis, allelopathy, and drought tolerance), and the utility of understanding the genetic and physiological basis of hyperaccumulation for technological purposes, a working definition for metal hyperaccumulation should be compatible with these pursuits (Morris et al., 2009; Boyd, 2013; Fones and Preston, 2013; Cappa and Pilon-Smits, 2014; Pollard et al., 2014). Current definitions of hyperaccumulation also confound tolerance with accumulation. Evidence strongly indicates that tolerance and accumulation are separate traits mediated by genetically and physiologically distinct mechanisms (Chaney et al., 1997; Hanikenne et al., 2008; Verbruggen et al., 2009), and may likewise possess distinct evolutionary trajectories. Accordingly, a physiological and evolutionary definition of hyperaccumulation should *not* include tolerance, as this would imply that the two traits are linked and mediated by the same mechanisms in accumulator and tolerant species. We argue that the separate mechanisms of hyperaccumulation and tolerance form two continuous axes producing four general categories: tolerant accumulator, non-tolerant accumulator, non-tolerant non-accumulator, and tolerant non-accumulator (Figure 1). Current conceptual definitions of hyperaccumulators fall within the first quadrant while excluding the second quadrant, and typically combine the third and fourth quadrants as non-hyperaccumulators (Figure 1). These definitions are therefore overly restrictive and are likely to result in the overlooking of evolutionarily relevant dynamics of tolerance and accumulation. Furthermore, when coupled with recommendations to only use natural soils, it becomes nearly impossible to disentangle tolerance from accumulation, as well as to identify facultative hyperaccumulators that have higher soil metal thresholds for significant uptake than found in the field. Restrictions on the use of amended soils are particularly incompatible with controlled phylogenetically explicit investigations, where fair comparisons among closely related species are typically achieved by being grown under uniform environmental conditions. Given that several sources recommend the use of phylogenetic assessments of multiple clades across the angiosperm phylogeny as the best method to examine a variety of aspects of hyperaccumulator evolution (van der Ent et al., 2013; Cappa and Pilon-Smits, 2014; Pollard et al., 2014), this restriction cannot stand.

We advocate the study of hyperaccumulation and tolerance in greenhouse studies by proposing the following guidelines:

- Tolerance and hyperaccumulation should be considered distinct continuous traits, and experiments should be designed to estimate both traits without confounding.
- Hyperaccumulation should be defined as the ability to produce leaf metal concentrations above the metal-specific criterion levels in the context of adequate soil metal concentrations.
- Tolerance should be based on physiological and reproductive performance traits. These traits will likely need to differ by system (e.g., survival, biomass, fecundity), but ideally plants should be grown to reproduction and assessed for production of viable offspring.
- For single-level experiments, metal concentrations in amended soils should be appropriately low enough to rule out passive hyperaccumulation but high enough to allow the detection of active hyperaccumulation. The use of soil amendments at multiple levels is more informative.
- Chelate-induced phytoextraction with mobilizing agents such as EDTA should not be used when screening for hyperaccumulation or tolerance, as such conditions artificially facilitate plant metal accumulation while buffering plants against toxicity, thus poorly reflecting both hyperaccumulation capacity and metal tolerance.

In addition to these guidelines, it is helpful to appreciate that the manifestation of both hyperaccumulation and tolerance are environment-dependent, and evaluations in a single environment (i.e., one soil metal concentration) are unlikely to capture the complex nature of these traits and their interaction. Greenhouse studies applying continuous gradients of metal concentrations via amended soils can be used to determine the extent of the dependence of hyperaccumulation on soil metal concentrations while simultaneously assessing metal tolerance. In this way, the traits of tolerance and hyperaccumulation can be more fully expressed as concentration-response curves. Phylogenetically explicit studies can further reveal whether tolerance and hyperaccumulation evolve together or sequentially, and provide a controlled framework for assessing the myriad adaptive hypotheses that have been put forward to explain this fascinating phenomenon.

### Conflict of interest statement

The authors declare that the research was conducted in the absence of any commercial or financial relationships that could be construed as a potential conflict of interest.
